# Supplementary material for: Femoral head collapse after hip intra-articular corticosteroid injection: An institutional response to improve practice and increase patient safety
Source: PLoS One. 2021 Nov 2;16(11):e0259242. doi: 10.1371/journal.pone.0259242 (PMC8562809; doi:10.1371/journal.pone.0259242)
Supplement: Attachment — Submitted filename: Response to Reviewers.docx [file pone.0259242.s001.docx]

**Response to Reviewer Comments**

**PONE-D-21-00877R1**

Reviewer 4:

1) Line 93, the authors reported: “witnessing a high rate” how much are they considered high? Is there a certain percentage they calculated, or data were reported from a previous survey or study in their institution?

Author Response:

At the end of 2017, we had observed and treated multiple patients who returned to clinic after hip intra-articular corticosteroid injection (IACSI) with debilitating symptoms secondary to femoral head collapse (FHC). Prior to our investigation, there was no known or previously reported rate of FHC at our institution. Additionally, there is a paucity of cases documented in the literature, and available case reports and series do not define any true incidence. However, FHC is considered a rare complication after IACSI. We have revised our manuscript as follows:

- **“****After witnessing multiple cases of FHC after hip IACSI, we reviewed and compared our experience and institutional care practices with evidence-based literature.” (*Materials & Methods, lines 93 - 94)***

Reviewer 4:

2) Line 116, the authors reported comparing the x-rays side by side; what was the exact tool used to judge the sphericity of the femoral head, how did they judge the proper rotation of the included radiographs? Did they exclude any cases due to bad-quality radiographs? Did the two residents performed check of the whole x-rays or these were split between them?

Author Response (part a):

Each radiograph was reviewed using the picture archiving and communication system (PACS). Pre- and post-injection radiographs were compared side by side for loss of contour secondary to collapse. For each hip image, a circle tool was used to outline the contour of the subchondral bone-edge in the weightbearing portion of the femoral head. We have added an explanation and clarification of methodology as follows:

- **“Post-injection FHC was defined radiographically as new loss of femoral head sphericity and was measured by comparing the contour of the weightbearing portion of the femoral head on each radiograph. For each hip image, a circle tool was used to outline the contour of the subchondral bone-edge in the weightbearing portion of the femoral head.” (*Materials & Methods, lines 117 -121)***

Author Response (part b):

The authors examined pre- and post-hip IACSI anterior-posterior (AP) pelvis or hip radiographs. Standard AP pelvis and/or hip radiographs were obtained. Per radiology department protocol, radiographs were reviewed by a radiology technician in real-time to ensure correct position and rotation is obtained. If a radiograph was inadequate a new radiograph was obtained prior to releasing the radiograph to PACS. There were no radiographs excluded due to malrotation. This point is highlighted in the manuscript as follows:

- **“****All radiographs were obtained using our institution’s standardized Radiology department protocol. No images were excluded for poor quality.” (*Materials & Methods, lines 114-116)***

Author Response (part c):

Each patient was reviewed independently by one of two orthopedic surgery residents. All patient radiographs then underwent confirmatory review by the senior author. This point is clarified in the manuscript as follows:

- **“Images were initially compared and interpreted by two orthopedic surgery residents and later independently verified by an attending orthopedic staff. In all cases, FHC was dramatic, easily detected, and the interim change in femoral head sphericity** **secondary to collapse was confirmed by official radiology reports.” (*Materials & Methods, lines 121-124)***

Reviewer 4:

3) Line 189. “Reduction in post-IACSI FHC was 67.9% (84 to 27)” was this reduction possibly due to reduced number of patients who had an injection? Or due to a relatively shorter time of follow up after implementing the quality improvement protocol?

Author Response:

The reviewer raises a very important point. We agree that, due to the shorter length of radiographic follow up for the post-intervention group, there is a possibility of bias impacting the results, falsely lowering the rate of FHC. While this is a weakness of our study, the possibility of a falsely low FHC rate in the post-intervention group strengthens our rationale for instituting the Clinical Pathway to standardize and study hip IACSI best practices. This weakness is acknowledged and addressed in the manuscript as follows:

- **“Due to the retrospective nature of this work, the radiographic follow-up for pre- and post-intervention study groups was non-standardized. Additionally, the time to chart/radiographic review was shorter for the post-intervention group, thereby introducing the potential for a falsely decreased FHC rate in the post-intervention group. While the shorter length of radiographic follow up for the post-intervention group and the possibility of a falsely low FHC rate in this group is a weakness of our study, it strengthens our rationale for instituting the Clinical Pathway to standardize hip IACSI practices so to allow for further prospective study.” (*Discussion, lines 351-357)***

Reviewer 4:

4) I would like the authors to add a statement in the acknowledgement thanking the residents for their effort if they were not listed as authors.

Author Response:

The residents responsible for the chart review are included as first and second authors.

Reviewer 5

1) I wonder whether the inclusion of AVN cases (8 cases) and given the possibility that the natural progression of the disease rather than the IACSI was the cause of the collapse rather than the IACSI??? I think this is worth elaborating upon specifically referring to the AVN cases please.

Author Response:

Avascular necrosis (AVN) or similar pathologies contributed to some of the FHCs observed at our institution. As this review was limited by its retrospective nature, we studied all patients referred for hip IACSI, including the 8 hips with a diagnosis of AVN. We have reported the diagnoses documented for all patients referred for hip IACSI as we felt it important to include all patients to best determine associated risk factors for FHC. Per the reviewer’s suggestions, we have added to the manuscript with additional information on the AVN cases as follows:

- **“There were 8 hips (1.51%) that had a diagnosis of avascular necrosis (AVN). One patient was excluded due to pre-existing FHC. Of the remaining 7 hips, 2 hips (2.38%) developed FHC, and 5 hips (1.52%) did not develop collapse after hip IACSI.” (*Results*, *lines 174 - 176*)**
- **“The natural history of avascular necrosis of the femoral head is FHC. In our analysis there were only 8 hips (1.51%) that had a diagnosis of AVN (7 ultimately included in analysis). Of the patients with AVN there was a 28.57% FHC rate (2/7) after IACSI. While there is a possibility AVN was underdiagnosed in our patient population, we were limited by the retrospective nature of the review and the information contained in the medical record at the time of retrieval. Due to the slightly higher FHC rate seen with this diagnosis and the natural history of the disease, we consider AVN as a relative contraindication for hip IACSI.” (*Discussion*, *lines 279 - 285*)**

Reviewer 5

2) Could you please elaborate more about the effect of the injection dose/material on the rate of collapse among those who have developed collapse and those who haven’t please?

Author Response:

The reviewer raises an excellent point. There is basic science evidence showing dose and volume-dependent chondrotoxicity with local anesthetics and corticosteroids. However, in our study, the variation in dosage and volume of hip injectate between providers was very substantial such that no association could be readily identified between FHC and the injectate. Ultimately, in response to this provider-to-provider variation and using the best available evidence to inform the safest medications and dosages for joint injections, the recommendation for the least toxic medications in the lowest effective dosage and volume were made. This point has been further clarified in the manuscript as below:

- **“No significant association was identified between FHC and local anesthetic or corticosteroid dosage, volume, or medication type.”** **(*Results*, *lines 188 - 190*)**
